# Relationship Between Pre-Pregnancy Body Mass Index, Gestational Weight Gain, and Perinatal Outcomes Among Women Delivering at a General Hospital, Nakhon Si Thammarat Province

**DOI:** 10.3390/ijerph23070924

**Published:** 2026-07-18

**Authors:** Sumoltip Sontimuang, Rachadaporn Jantasuwan, Suda Jaihow, Kanyapak Pluemjai, Luksamon Luksanavimon, Salwismawati Badrin, Hasni Embong

**Affiliations:** 1 Division of Pediatric Nursing, School of Nursing, The Excellence Center of Community Health Promotion, Walailak University, Nakhon Si Thammarat 80161, Thailand; poysontimuang@gmail.com; 2 Division of Community Nursing, School of Nursing, The Excellence Center of Community Health Promotion, Walailak University, Nakhon Si Thammarat 80161, Thailand; luksamon1965@gmail.com; 3 Division of Maternal-Newborn and Midwifery Nursing, School of Nursing, The Excellence Center of Community Health Promotion, Walailak University, Nakhon Si Thammarat 80161, Thailand; sudajaihow@gmail.com (S.J.); kanyapak.pl@wu.ac.th (K.P.); 4 Nursing Program, School of Health Sciences, Universiti Sains Malaysia, Health Campus, Kota Bharu 16050, Kelantan, Malaysia; salwis@usm.my (S.B.); ehasni@usm.my (H.E.)

**Keywords:** small for gestational age (SGA), small head circumference, fetal growth, maternal nutritional status

## Abstract

**Highlights:**

**Public health relevance—How does this work relate to a public health issue?**

- This study addresses maternal and child health issues, as pre-pregnancy nutritional status and gestational weight gain (GWG) are associated with neonatal health outcomes.
- Inappropriate pre-pregnancy body mass index (BMI) and inadequate or excessive GWG may increase the risk of adverse perinatal outcomes, contributing to public health burdens and reflecting the effectiveness of maternal healthcare services.

**Public health significance—Why is this work of significance to public health?**

- The findings provide important evidence regarding modifiable maternal nutritional risk factors that can help promote maternal health and reduce adverse perinatal outcomes.
- The results may support the development of surveillance measures, screening strategies, nutritional counseling, and weight management programs for women of reproductive age and pregnant women.

**Public health implications—What are the key implications or messages for practitioners, policymakers and/or researchers in public health?**

- Healthcare providers should promote pre-pregnancy BMI assessment, encourage optimal nutritional status among women of reproductive age, and continuously monitor GWG according to recommended guidelines to prevent maternal and perinatal complications.
- Policymakers and researchers should develop context-specific nutritional promotion and maternal care strategies tailored to the Thai population in order to improve long-term maternal and perinatal health outcomes.

**Abstract:**

This retrospective study examined the associations between pre-pregnancy body mass index (BMI), gestational weight gain (GWG), and perinatal outcomes among women with singleton pregnancies delivering at a general hospital in Nakhon Si Thammarat Province, Thailand. Secondary data were retrieved from the HosXP electronic medical database and delivery records. Of 4659 eligible delivery records, 4051 women met the study criteria and were included in the analysis. Multivariable binary logistic regression models were used to estimate adjusted odds ratios (aORs) and 95% confidence intervals (CIs). The incidences of preterm birth, small-for-gestational-age (SGA) infants, and below-optimal head circumference (HC) were 6.99%, 18.22%, and 42.85%, respectively. Maternal underweight before pregnancy was significantly associated with increased odds of preterm birth (aOR = 1.60, 95% CI: 1.21–2.12), SGA (aOR = 1.79, 95% CI: 1.44–2.24), and below-optimal HC (aOR = 1.44, 95% CI: 1.18–1.76). Inadequate GWG was also associated with higher odds of SGA (aOR = 1.66, 95% CI: 1.38–2.00) and below-optimal HC (aOR = 1.29, 95% CI: 1.11–1.49). Conversely, maternal overweight/obesity and excessive GWG were associated with lower odds of SGA and below-optimal HC. These findings indicate that pre-pregnancy BMI and GWG were independently associated with adverse perinatal outcomes among Thai women with singleton pregnancies and may provide context-specific evidence to support maternal nutritional assessment and gestational weight monitoring during antenatal care.

## 1. Introduction

Maternal nutritional status before and during pregnancy is a major determinant of perinatal health and represents an important and potentially modifiable public health factor. Pre-pregnancy body mass index (BMI) is widely used to assess maternal nutritional status and reflects both undernutrition and overnutrition, conditions that may adversely affect maternal well-being and fetal growth. Both extremes of BMI have been associated with increased risks of perinatal outcomes, highlighting the importance of optimal maternal weight prior to conception. Globally, the mean BMI of women of reproductive age has increased steadily over recent decades, driven by changes in dietary patterns, reduced physical activity, and broader socioeconomic transitions. Data from the World Health Organization (WHO) and related epidemiological studies indicate a sustained upward trend in BMI since the 1980s [1]. In the United States, the prevalence of pre-pregnancy obesity nearly doubled between the early 1990s and 2010, significantly influencing perinatal health indicators [1]. More recent estimates (2017–2023) demonstrate rising obesity prevalence not only in high-income countries such as the United States, the United Kingdom, and Australia but also in middle-income settings, including Brazil and China [2]. At the same time, underweight remains prevalent in certain populations, creating a dual burden of malnutrition.

Pre-pregnancy BMI is associated with a range of perinatal outcomes [3]. Among pregnant women from Asian-American ethnic groups, maternal underweight status was associated with an increased likelihood of delivering smallforgestational-age (SGA) infants compared with women with normal BMIs. Specifically, underweight mothers demonstrated 1.37 higher odds of SGA birth outcomes (aOR = 1.37; 95% CI: 1.30–1.46) [4]. In addition, these conditions may influence fetal brain and head development through epigenetic mechanisms [5]. Conversely, evidence from a meta-analysis including 86 studies and more than 20 million pregnant women indicated that maternal overweight and obesity were associated with a higher likelihood of several adverse maternal and perinatal outcomes. These included cesarean delivery (both elective and emergency), gestational diabetes mellitus (GDM), gestational hypertension, labor induction, postpartum hemorrhage, preeclampsia, and preterm premature rupture of membranes. In addition, infants born to overweight or obese mothers were more likely to require admission to a neonatal intensive care unit (NICU) [6].

Gestational weight gain (GWG), which is partly determined by pre-pregnancy BMI, represents an additional modifiable factor influencing perinatal outcomes. Both inadequate and excessive GWG are associated with adverse consequences. A recent meta-analysis demonstrated that inadequate GWG was associated with an increased risk of adverse perinatal outcomes in a dose-dependent manner. Compared with adequate GWG, moderately inadequate GWG was associated with higher risks of low birth weight (LBW) (aRR = 1.26, 95% CI: 1.20–1.32), SGA (aRR = 1.22, 95% CI: 1.18–1.26), and microcephaly (aRR = 1.26, 95% CI: 1.12–1.41). The risks were greater among women with severely inadequate GWG, including LBW (aRR = 1.62, 95% CI: 1.51–1.72), SGA (aRR = 1.44, 95% CI: 1.36–1.54), and microcephaly (aRR = 1.57, 95% CI: 1.31–1.88) [7]. Conversely, excessive GWG has been associated with an increased risk of macrosomia and cesarean delivery, while maternal obesity increases the likelihood of GDM, hypertensive disorders of pregnancy, fetal distress, postpartum hemorrhage, and preterm birth [8]. The Institute of Medicine (IOM) has established recommendations for GWG based on pre-pregnancy BMI. Women classified as underweight (BMI < 18.5 kg/m^2^) are advised to gain 12.5–18 kg during pregnancy, whereas those with normal BMI (18.5–24.9 kg/m^2^) should gain 11.5–16 kg. The recommended GWG for overweight women (BMI 25.0–29.9 kg/m^2^) is 7–11.5 kg, while women with obesity (BMI ≥ 30 kg/m^2^) are advised to gain 5–9 kg throughout pregnancy [9]. Previous evidence has shown that GWG below the recommended range was associated with a higher risk of intrauterine growth restriction (IUGR) (aOR = 1.61; 95% CI: 1.14–2.27), LBW (aOR = 2.44; 95% CI: 1.85–3.21), and preterm birth (aOR = 2.35; 95% CI: 1.81–3.05). In contrast, inadequate GWG was associated with a reduced likelihood of large for gestational age (LGA) infants (aOR = 0.38; 95% CI: 0.28–0.54). Conversely, excessive GWG increased the risk of LGA birth outcomes by 1.53 times (aOR = 1.53; 95% CI: 1.20–1.96) [2].

In addition, underweight is associated with fetal growth restriction and preterm delivery. However, some evidence suggests that excessive GWG among women with high pre-pregnancy BMI is more consistently associated with fetal overgrowth than with preterm birth [10], indicating heterogeneity across outcomes. Appropriate adherence to these guidelines has been shown to improve maternal and perinatal outcomes.

In Thailand, rapid lifestyle transitions—including increased consumption of energy-dense and ultra-processed foods—have contributed to rising obesity among women aged 15–29 years, while underweight remains prevalent among adolescents [11]. This dual burden may amplify risks at both extremes of the BMI distribution and underscores the need for locally generated evidence to inform maternal health strategies.

Although numerous studies have examined the associations between pre-pregnancy BMI, GWG, and perinatal outcomes, most evidence has been generated in high-income countries. The applicability of these findings to Thailand remains uncertain because maternal nutritional status, dietary patterns, healthcare practices, and population characteristics differ substantially across settings. Thailand is currently experiencing a dual burden of malnutrition, with the coexistence of maternal underweight and obesity among women of reproductive age. These unique population characteristics may modify the relationships between maternal anthropometry and perinatal outcomes. Moreover, evidence from Thailand remains limited, particularly studies evaluating both pre-pregnancy BMI and GWG in relation to multiple perinatal outcomes within the same population. Therefore, this study aimed to examine the relationship between pre-pregnancy BMI, GWG, and perinatal outcomes among women delivering at a general hospital in Thailand. The findings are expected to provide context-specific evidence to support antenatal nutritional counseling, gestational weight monitoring, and public health strategies aimed at improving maternal and perinatal health outcomes.

## 2. Materials and Methods

### 2.1. Research Design and Participants

This retrospective study aimed to investigate the associations between pre-pregnancy BMI, GWG, and perinatal outcomes among women who delivered at a general hospital in Nakhon Si Thammarat Province, Thailand. Data were retrieved from the HosXP electronic medical database and delivery records of 4659 pregnant women who gave birth between 1 October 2020 and 30 September 2023. The researchers obtained permission to use the data from the principal investigator of the research project entitled “Factors related to anemia and birth outcomes of women attending antenatal care at Thasala Hospital, Nakhon Si Thammarat Province.”

### 2.2. Inclusion and Exclusion Criteria

Participants were eligible for inclusion if they (1) were of Thai nationality; (2) had a singleton pregnancy; (3) had a documented pre-pregnancy BMI; (4) had recorded pre-pregnancy weight; and (5) had a recorded last measured weight prior to delivery. Cases were excluded if (1) the infant had congenital hydrocephalus and (2) perinatal birth outcome data were incomplete.

### 2.3. Research Instruments

The researchers obtained permission to use data from the research project entitled “Factors related to anemia and birth outcomes of women attending antenatal care at Thasala Hospital, Nakhon Si Thammarat Province.” The research instruments consisted of three components: (1) a demographic data record form, including maternal age at delivery, educational level, and religion; (2) a maternal health record form during pregnancy, including gravidity, history of abortion, gestational age (GA) at first antenatal care (ANC) visit, number of quality ANC visits, type of delivery, underlying diseases, pre-pregnancy BMI, and GWG; and (3) a perinatal outcomes record form, including GA at delivery, birth weight by gestational age, and HC. A standardized data extraction form was developed based on the study variables and the hospital electronic medical record system. Prior to data collection, the form was pilot-tested using a sample of medical records to ensure the completeness, clarity, and consistency of data extraction. GWG was calculated as the difference between maternal weight measured before delivery and pre-pregnancy weight. GWG was subsequently categorized as below, optimal, or above the recommended range according to the IOM recommendations for each pre-pregnancy BMI category [9].

This study classified birth weight by GA according to the INTERGROWTH-21st standards, which categorize perinatal birth weight into three groups: SGA, defined as birth weight below the 10th percentile; appropriate for gestational age (AGA), defined as birth weight between the 10th and 90th percentiles; and LGA, defined as birth weight above the 90th percentile [12]. Neonatal sex was unavailable in the secondary dataset. HC among preterm infants (<37 weeks of gestation) was classified using the Fenton Preterm Growth Chart, which updated the Babson and Benda fetal–infant growth chart. This reference was developed using averaged data from male and female infants to generate a single set of percentile curves; therefore, neonatal sex was not required for HC classification. HC values between the 10th and 90th percentiles were classified as optimal, values below the 10th percentile as below-optimal HC, and values above the 90th percentile as above-optimal HC [13]. For full-term infants (≥37 weeks of gestation), HC was classified according to the National Maternity Data Development Project: Baby Head Circumference reference for healthy full-term infants, which likewise provides a single reference based on the combined average of male and female newborns. Accordingly, an HC of 33–37 cm at birth was classified as optimal HC, whereas HC < 33 cm and HC > 37 cm were classified as below-optimal HC and above-optimal HC, respectively [14].

### 2.4. Data Analysis

Descriptive statistics were used to analyze participants’ baseline characteristics and are presented as frequencies and percentages. Associations between pre-pregnancy BMI, GWG, and perinatal outcomes were examined using the chi-square test or Fisher’s exact test, as appropriate. Multivariable binary logistic regression models were used to examine the associations of pre-pregnancy BMI and GWG with perinatal outcomes. Pre-pregnancy BMI and GWG were entered simultaneously into each model using the enter method; no additional covariates were included. Thus, the association of each exposure with the outcome was estimated while accounting for the other exposure included in the model, and adjusted odds ratios (aORs) with 95% confidence intervals (CIs) were reported. Separate models were constructed for each perinatal outcome: GA at delivery, birth weight by GA, and neonatal HC. For GA at delivery, term birth (≥37 weeks) was coded as 0 and preterm birth (<37 weeks) as 1. For birth weight by GA, AGA and LGA were combined and coded as 0, whereas SGA was coded as 1. For perinatal HC, optimal and above-optimal HC were combined and coded as 0, whereas below-optimal HC was coded as 1. Categorical exposure variables were entered using indicator (dummy) coding, with the first category designated as the reference group.

Previous methodological studies using Monte Carlo simulation have suggested that logistic regression models should include at least 10 events per predictor variable (EPV) to ensure stable and reliable parameter estimation [15]. The sample size in the present study was considered adequate to support the validity and stability of the regression models.

### 2.5. Ethical Consideration

The research project was approved by the Committee on Human Rights Related to Research Involving Human Subjects, Walailak University, Thailand (Approval No. WUEC-24-304-02).

## 3. Results

### 3.1. Participant Characteristics

A total of 4659 women with singleton pregnancies were initially identified from the electronic medical database. After applying the predefined inclusion and exclusion criteria, 4051 women were eligible and included in the final analysis. The incidence of preterm birth was 6.99%, SGA 18.22%, and small HC 42.85%. Most participants were of maternal age 20–34 years (73.86%), while pregnant adolescents accounted for 8.00% and women of advanced maternal age represented 18.14%. The most common mode of delivery was cesarean section, accounting for 48.04% of all deliveries.

Regarding educational attainment, the majority had completed secondary school or equivalent education (58.46%), followed by those holding a bachelor’s degree or higher (22.83%). Most participants identified as Buddhist (74.65%). Maternal age was significantly associated with GA at delivery (*p* = 0.015), birth weight by GA (*p* < 0.001), and HC (*p* < 0.001). Type of delivery was significantly associated with birth weight by GA and HC (both *p* < 0.001). Educational level showed a significant association with HC (*p* = 0.003), whereas religion was significantly associated with GA at delivery (*p* = 0.013), as presented in Table 1.

### 3.2. Factors Associated with Perinatal Outcomes

Regarding factors associated with perinatal outcomes, gravidity was significantly associated with birth weight by gestational age and HC (all *p* < 0.001). A history of abortion was significantly related to GA at delivery, birth weight by GA, and HC (*p* < 0.001, *p* = 0.005, and *p* = 0.017, respectively). GA at the first ANC visit was significantly associated with birth weight by GA (*p* = 0.012). The number of quality ANC visits showed significant associations with GA at delivery (*p* < 0.001). In addition, underlying diseases were significantly associated with GA at delivery, birth weight by GA, and HC (*p* < 0.001, *p* = 0.001, and *p* = 0.031, respectively). Pre-pregnancy BMI demonstrated significant associations with birth weight by GA and HC (both *p* < 0.001). Similarly, GWG was significantly associated with GA at delivery, birth weight by GA, and HC (all *p* < 0.001), as shown in Table 2.

### 3.3. Multivariable Associations of Pre-Pregnancy BMI and GWG with Perinatal Outcomes

Multivariable binary logistic regression analysis was performed to identify factors associated with perinatal outcomes. The findings indicated that mothers with pre-pregnancy underweight were at significantly higher risk of preterm birth (aOR = 1.60; 95% CI: 1.21–2.12), SGA infants (aOR = 1.79; 95% CI: 1.44–2.24), and below-optimal HC (aOR = 1.44; 95% CI: 1.18–1.76). In contrast, maternal overweight/obesity was associated with lower odds of SGA (aOR = 0.64, 95% CI: 0.52–0.78) and below-optimal HC (aOR = 0.70, 95% CI: 0.61–0.80).

Inadequate GWG was significantly associated with an increased risk of SGA (aOR = 1.66; 95% CI: 1.38–2.00) and below-optimal HC (aOR = 1.29; 95% CI: 1.11–1.49). Conversely, excessive GWG appeared to reduce the risk of SGA (aOR = 0.74; 95% CI: 0.58–0.95) and below-optimal HC (aOR = 0.75; 95% CI: 0.63–0.90), as presented in Table 3.

Overall, both pre-pregnancy underweight status and inadequate GWG were strongly associated with impaired fetal growth, whereas GWG showed no significant relationship with preterm birth.

## 4. Discussion

In this retrospective study involving 4051 women with singleton pregnancies, we investigated the associations between pre-pregnancy BMI, GWG, and adverse perinatal outcomes. The multivariable binary logistic regression analyses yielded two major findings regarding pre-pregnancy BMI.

First, maternal underweight status before pregnancy was independently associated with increased odds of preterm birth, SGA infants, and below-optimal HC. These findings are consistent with previous studies reporting that pre-pregnancy underweight is associated with adverse fetal growth outcomes, including preterm birth, SGA, and reduced HC [16,17,18]. Similar associations between maternal underweight and SGA have also been reported in several recent studies [19,20,21]. Several biological mechanisms may explain the observed associations. It is possible that low maternal BMI is associated with impaired uteroplacental circulation and placental development, resulting in suboptimal villous structure and reduced placental efficiency [16,22]. Consequently, the transfer of essential nutrients, particularly glucose and amino acids, to the fetus may be reduced, which may contribute to fetal growth restriction (FGR), SGA birth, impaired brain development, and smaller neonatal HC [23]. Furthermore, inadequate fetal growth may activate the fetal hypothalamic–pituitary–adrenal (HPA) axis, leading to increased secretion of cortisol and corticotropin-releasing hormone (CRH). These hormonal alterations may contribute to the initiation of preterm labor [24,25].

Second, maternal overweight and obesity were associated with lower odds of SGA and below-optimal HC, the fetal growth outcomes evaluated in the present study. These findings are consistent with previous evidence indicating that maternal overweight and obesity are associated with an increased tendency toward LGA infants [26,27]. Several biological mechanisms may explain these findings. Maternal overweight and obesity are associated with increased insulin resistance and elevated circulating levels of insulin, leptin, insulin-like growth factor-1 (IGF-1), and lipids, which may activate the placental mechanistic target of rapamycin (mTOR) signaling pathway and enhance placental nutrient transport to the fetus [28]. In addition, the syncytiotrophoblast can sense changes in maternal nutrient availability and adapt placental structure and function, thereby regulating fetal nutrient supply and promoting fetal growth. Excess maternal nutrient exposure may overstimulate these nutrient-sensing pathways, resulting in accelerated fetal growth and an increased tendency toward fetal overgrowth [29]. However, these findings should be interpreted only in relation to the specific fetal growth outcomes examined in this study and should not be considered evidence of an overall beneficial effect of maternal overweight or obesity during pregnancy. Previous studies have shown that maternal obesity is associated with placental inflammation, oxidative stress, and lipid accumulation, which may increase the long-term risk of metabolic disorders in offspring [22,28]. In addition, excess maternal adiposity may promote placental inflammation and oxidative stress, leading to placental dysfunction and increasing the risks of macrosomia, shoulder dystocia, cesarean delivery, perinatal hypoglycemia, and structural fetal defects [22,26,30]. Furthermore, pre-pregnancy obesity has been associated with increased placental thickness and impaired placental function, which may reduce oxygen diffusion to the fetus and subsequently increase the risk of perinatal distress and stillbirth [16].

With regard to GWG, inadequate GWG had higher odds of delivering SGA infants and infants with below-optimal HC, whereas those with excessive GWG showed lower odds of these outcomes. It is important to note that these findings are specific to the fetal growth indicators examined in the present study and should not be interpreted as suggesting that excessive GWG is advantageous during pregnancy, given its well-established associations with other adverse maternal and perinatal outcomes. Regarding GWG, inadequate GWG was identified as a significant predictor of SGA and below-optimal HC. These findings are consistent with previous studies demonstrating that inadequate GWG is associated with impaired fetal growth outcomes, particularly SGA [21] and reduced HC [7]. In addition, insufficient GWG has been reported to increase the risk of LBW infants [17,31,32]. Several biological mechanisms may explain these associations. Inadequate GWG may reflect insufficient maternal nutritional reserves, which can impair placental growth and reduce uteroplacental perfusion [31,33]. Consequently, the delivery of essential nutrients, including glucose, amino acids, and fatty acids, to the fetus may be compromised, contributing to fetal growth restriction (FGR), SGA, and impaired brain development [34,35,36]. Furthermore, reduced levels of insulin and insulin-like growth factor-1 (IGF-1) may further inhibit cellular proliferation and fetal organ development [16,35,37].

Our findings also showed that excessive GWG was associated with lower odds of SGA and below-optimal HC. This observation should be interpreted within the context of fetal growth outcomes and does not imply that excessive GWG is favorable for overall maternal or perinatal health. This observation is supported by a meta-analysis of 47 studies reporting that excessive GWG increased the risk of LGA and macrosomia compared with adequate GWG, reflecting enhanced fetal growth among mothers with excessive weight gain during pregnancy [30]. This finding may be explained by the fact that excessive GWG tends to result in infants with greater perinatal fat mass and higher body fat percentage. Increased nutrient availability in the maternal circulation may stimulate placental nutrient transport, thereby promoting fetal overgrowth [16,36]. Consequently, enhanced fetal growth may reduce the likelihood of SGA and below-optimal HC [38].

However, excessive GWG has also been linked to several adverse maternal and perinatal outcomes, including macrosomia, shoulder dystocia, and increased cesarean delivery rates [7,26,38,39,40]. Several biological mechanisms may explain these associations. Maternal overnutrition may induce chronic inflammation and oxidative stress, which can impair placental structure and function [18,22,41]. These metabolic disturbances may subsequently increase the risk of perinatal hypoglycemia and long-term metabolic disorders in offspring. Furthermore, placental dysfunction associated with maternal overnutrition may contribute to fetal hypoxia and stillbirth [16,22].

The prevalence of below-optimal HC in the present study was 42.85%, which was considerably higher than the reported prevalence of microcephaly (11.60–16.00%) in a previous retrospective study of 121,448 newborns conducted between 2014 and 2018 [42]. However, this comparison should be interpreted with caution because different HC classification criteria were applied. The previous study classified neonatal HC using the updated INTERGROWTH-21st standards for term newborns and the revised Fenton growth chart for preterm infants, both of which incorporate sex-specific reference standards. In contrast, neonatal sex was unavailable in the present secondary dataset, and HC was classified using non-sex-specific reference criteria. Moreover, the HC reference standards applied in the present study were developed from populations outside Thailand and may not fully reflect the anthropometric characteristics of Thai newborns. These methodological differences may have contributed, at least in part, to the higher prevalence of below-optimal HC observed in the present study. Furthermore, the relatively high prevalence of third-trimester anemia (21.44%), pre-pregnancy underweight (12.02%), and inadequate GWG (42.34%) in the study population may also have contributed to the high proportion of below-optimal HC observed in the present study, as these maternal conditions have previously been associated with impaired fetal growth and fetal head development.

This study underscores the importance of preparing women of reproductive age to achieve an appropriate BMI prior to conception, as optimal pre-pregnancy nutritional status may help reduce the risk of adverse perinatal outcomes, including preterm birth, SGA infants, and below-optimal HC. In addition, achieving GWG within recommended ranges appears to play a crucial role in minimizing unfavorable pregnancy outcomes. Integrating maternal weight management into routine ANC services is therefore essential. Early assessment of maternal BMI during the first ANC visit provides a valuable opportunity to identify women at increased risk of adverse pregnancy outcomes and to initiate timely, individualized interventions. Furthermore, adherence to established GWG recommendations, together with the promotion of healthy lifestyle behaviors—such as balanced nutrition, appropriate physical activity, and continuous weight monitoring throughout pregnancy—may help mitigate the risks associated with both inadequate and excessive GWG. Collectively, these strategies may contribute to improved maternal and perinatal health outcomes.

## 5. Strengths and Limitations

This study has several strengths. It included a large sample of women with singleton pregnancies and simultaneously examined the associations of pre-pregnancy BMI and gestational weight gain with multiple perinatal outcomes. The use of routinely collected electronic medical records also minimized recall bias and reflected real-world clinical practice.

However, several limitations should be acknowledged. First, the retrospective observational design precludes causal inference between maternal anthropometric factors and perinatal outcomes. Second, this study relied on secondary data extracted from electronic medical records; therefore, several potentially important variables, including maternal dietary intake, physical activity, smoking status, socioeconomic characteristics, pregnancy complications, adherence to iron supplementation, and neonatal sex, were unavailable for analysis. Consequently, residual confounding cannot be excluded. Third, because neonatal sex was unavailable, sex-specific reference charts could not be applied when classifying neonatal HC. Although published reference standards appropriate for preterm and full-term infants were used, the absence of neonatal sex information may have resulted in some degree of misclassification of HC, potentially leading to an overestimation of the prevalence of below-optimal HC. Fourth, the study was conducted at a single general hospital in southern Thailand, which may limit the generalizability of the findings to other settings or populations. Finally, GWG was calculated using pre-pregnancy weight and maternal weight recorded before delivery. Although this approach is commonly used in retrospective studies, measurement errors related to routine clinical records may have occurred.

## 6. Conclusions

In this retrospective study of Thai women with singleton pregnancies delivering at a general hospital, we examined the associations of pre-pregnancy BMI and GWG with selected perinatal outcomes. The multivariable binary logistic regression analyses showed that maternal underweight and inadequate GWG were associated with higher odds of SGA and below-optimal HC, while maternal underweight was also associated with preterm birth. Maternal overweight/obesity and excessive GWG were associated with lower odds of SGA and below-optimal HC. However, these associations were observed only for the specific fetal growth outcomes evaluated in this study and should not be interpreted as evidence that maternal overweight/obesity or excessive GWG is beneficial during pregnancy overall. These findings highlight the importance of maternal nutritional status before and during pregnancy and provide context-specific evidence regarding the associations of pre-pregnancy BMI and GWG with perinatal outcomes among Thai women. Further prospective studies are warranted to confirm these findings and to determine whether optimizing maternal nutritional status before and during pregnancy improves maternal and perinatal health outcomes.

## Figures and Tables

**Table 1:** General characteristic of participants (n = 4051) stratified by GA at delivery, birth weight, and size for gestational age.

General Characteristic	Overall	GA at Delivery (Wks)				Birth Weight by GA					HC				
		<37 n (%)	≥37 n (%)	χ^2^/^F^	*p*-Value	SGA n (%)	AGA n (%)	LGA n (%)	χ^2^/^F^	*p*-Value	Below n (%)	Optimal n (%)	Above n (%)	χ^2^/^F^	*p*-Value
	4051 (100.00)	283 (6.99)	3768 (93.01)			738 (18.22)	3181 (78.52)	132 (3.26)			1736 (42.85)	2267 (55.96)	48 (1.18)		
**Age (Year)**				8.41	0.015 *				40.45	<0.001 ***				52.02	<0.001 ***
≤19	324 (8.00)	26 (8.02)	298 (91.98)			91 (28.09)	232 (71.60)	1 (0.31)			194 (59.88)	128 (39.51)	2 (0.61)		
20–34	2992 (73.86)	189 (6.32)	2803 (93.68)			530 (17.71)	2370 (79.22)	92 (3.07)			1271 (42.48)	1689 (56.45)	32 (1.07)		
≥35	735 (18.14)	68 (9.25)	667 (90.75)			117 (15.92)	579 (78.77)	39 (5.31)			271 (36.87)	450 (61.23)	14 (1.90)		
**Type of delivery**				2.65	0.264				60.89	<0.001 ***				336.12	<0.001 ***
Normal Labor	2047 (50.53)	137 (6.69)	1910 (93.31)			449 (21.93)	1560 (76.21)	38 (1.86)			1159 (56.62)	872 (42.60)	16 (0.78)		
Cesarean Section	1946 (48.04)	139 (7.14)	1807 (92.86)			281 (14.44)	1572 (80.78)	93 (4.78)			546 (28.06)	1368 (70.30)	32 (1.64)		
Others	58 (1.43)	7 (12.07)	51 (87.93)			8 (13.79)	49 (84.48)	1 (1.73)			31 (53.45)	27 (46.55)	0(0.00)		
**Educational Level**				2.48	0.48				12.04	0.061				20.23	0.003 **
Below Secondary School	500 (12.34)	33 (6.60)	467 (93.40)			106 (21.20)	383 (76.60)	11 (2.20)			247 (49.40)	249 (49.80)	4 (0.80)		
Secondary School or Equivalent	2368 (58.46)	177 (7.47)	2191 (92.53)			448 (18.92)	1838 (77.62)	82 (3.46)			1032 (43.58)	1306 (55.15)	30 (1.27)		
Diploma/Associate Degree	258 (6.37)	14 (5.43)	244 (94.57)			45 (17.44)	205 (79.46)	8 (3.10)			102 (39.53)	151 (58.53)	5 (1.94)		
Bachelor’s Degree or Higher	925 (22.83)	59 (6.38)	866 (93.62)			139 (15.03)	755 (81.62)	31 (3.35)			355 (38.38)	561 (60.65)	9 (0.97)		
**Religion**				8.66 ^F^	0.013 *				2.00 ^F^	0.816				4.603	0.100
Buddhism	3024 (74.65)	231 (7.64)	2793 (92.36)			543 (17.96)	2382 (78.77)	97 (3.21)			1285 (42.49)	1697 (56.12)	42 (1.39)		
Islam	1027 (25.35)	52 (5.06)	975 (94.94)			195 (18.99)	797 (77.60)	35 (3.41)			451 (43.91)	570 (55.50)	6 (0.59)		

χ^2^ = Chi-square test; ^F^ = Fisher’s exact test, * *p* < 0.05, ** *p* < 0.01, *** *p* < 0.001.

**Table 2:** Factors associated with perinatal outcomes.

Related Factors	Overall	GA at Delivery (Wks)				Birth Weight by GA					HC				
		<37 n (%)	≥37 n (%)	χ^2^/^F^	*p*-Value	SGA n (%)	AGA n (%)	LGA n (%)	χ^2^/^F^	*p*-Value	Below n (%)	Optimal n (%)	Above n (%)	χ^2^/^F^	*p*-Value
	4051 (100.00)	283 (6.99)	3768 (93.01)			738 (18.22)	3181 (78.52)	132 (3.26)			1736 (42.85)	2267 (55.96)	48 (1.18)		
**Gravidity**				4.71	0.094				60.75	<0.001 ***				60.65	<0.001 ***
Primigravida	1206 (29.77)	88 (7.30)	1118 (92.70)			296 (24.54)	888 (73.63)	22 (1.83)			608 (50.41)	592 (49.09)	6 (0.50)		
Gravida 2	1288 (31.79)	74 (5.75)	1214 (94.25)			225 (17.47)	1019 (79.11)	44 (3.42)			562 (43.63)	705 (54.74)	21 (1.63)		
Gravida ≥ 3	1557 (38.44)	121 (7.77)	1436 (92.23)			217 (13.94)	1274 (81.82)	66 (4.24)			566 (36.35)	970 (62.30)	21 (1.35)		
**History of abortion**				23.92	<0.001 ***				14.80	0.005 **				12.09	0.017 *
None	3247 (80.15)	206 (6.34)	3041 (93.66)			622 (19.15)	2529 (77.89)	96 (2.96)			1428 (43.98)	1782 (54.88)	37 (1.14)		
1 time	668 (16.49)	54 (8.08)	614 (91.92)			92 (13.77)	547 (81.89)	29 (4.34)			250 (37.43)	407 (60.93)	11 (1.64)		
≥2 times	136 (3.36)	23 (16.91)	113 (83.09)			24 (17.65)	105 (77.21)	7 (5.14)			58 (42.65)	78 (57.35)	0(0.00)		
**GA at first ANC**				0.40	0.529				8.85	0.012 *				2.69	0.260
≤12 wks	2405 (59.37)	163 (6.78)	2242 (93.22)			405 (16.84)	1914 (79.58)	86 (3.58)			1007 (41.87)	1367 (56.84)	31 (1.29)		
>12 wks	1646 (40.63)	120 (7.29)	1526 (92.71)			333 (20.23)	1267 (76.97)	46 (2.80)			729 (44.29)	900 (54.68)	17 (1.03)		
**Number of quality ANC**				50.29	<0.001 ***				0.87	0.646				2.87	0.238
<8 times	717 (17.70)	94 (13.11)	623 (86.89)			124 (17.29)	572 (79.78)	21 (2.93)			327 (45.61)	383 (53.42)	7 (0.97)		
≥8 times	3334 (40.63)	189 (5.67)	3145 (94.33)			614 (18.42)	2609 (78.25)	111 (3.33)			1409 (42.26)	1884 (56.51)	41 (1.23)		
**Underlying diseases**				33.57 ^F^	<0.001 ***				22.70 ^F^	0.001 **				13.88 ^F^	0.031 *
None	3556 (87.78)	223 (6.27)	3333 (93.73)			653 (18.36)	2796 (78.63)	107 (3.01)			1552 (43.64)	1965 (55.26)	39 (1.10)		
Overt DM/GDM	340 (8.39)	41 (12.06)	299 (87.94)			48 (14.12)	268 (78.82)	24 (7.06)			115 (33.82)	219 (64.41)	6 (1.76)		
HTN/PIH	55 (1.36)	13 (23.64)	42 (76.36)			14 (25.46)	40 (72.73)	1 (1.82)			24 (43.64)	30 (54.55)	1 (1.81)		
Others	100 (2.47)	6 (6.00)	94 (94.00)			23 (23.00)	77 (77.00)	0(0.00)			45 (45.00)	53 (53.00)	2 (2.00)		
**Pre-pregnancy BMI**				1.80	0.407				131.64	<0.001 ***				77.17	<0.001 ***
Underweight	487 (12.02)	41 (8.42)	446 (91.58)			151 (31.01)	331 (67.97)	5 (1.02)			269 (55.24)	216 (44.35)	2 (0.41)		
Normal weight	2097 (51.76)	144 (6.87)	1953 (93.13)			414 (19.74)	1639 (78.16)	44 (2.10)			956 (45.59)	1118 (53.31)	23 (1.10)		
Overweight/Obesity	1467 (36.22)	98 (6.68)	1369 (93.32)			173 (11.79)	1211 (82.55)	83 (5.66)			511 (34.83)	933 (63.60)	23 (1.57)		
**GWG**				23.404	<0.001 ***				113.50	<0.001 ***				67.46	<0.001 ***
Below	1715 (42.34)	157 (9.15)	1558 (90.85)			422 (24.61)	1264 (73.70)	29 (1.69)			846 (49.33)	850 (49.56)	19 (1.11)		
Optimal	1347 (33.25)	81 (6.01)	1266 (93.99)			209 (15.52)	1091 (80.99)	47 (3.49)			561 (41.65)	771 (57.24)	15 (1.11)		
Above	989 (24.41)	45 (4.55)	944 (95.45)			107 (10.82)	826 (83.51)	56 (5.66)			329 (33.27)	646 (65.32)	14 (1.41)		

χ^2^ = Chi-square test; ^F^ = Fisher’s exact test, * *p* < 0.05, ** *p* < 0.01, *** *p* < 0.001.

**Table 3:** Associations of BMI and GWG with perinatal outcomes using multivariable binary logistic regression (n = 4051).

Factors	Overall	GA at Delivery (Wks)				Birth Weight by GA				HC			
		Preterm	Term	aOR (95% CI)	*p*-Value	SGA	AGA/LGA	aOR (95% CI)	*p*-Value	Below	Optimal/Above	aOR (95% CI)	*p*-Value
	n (%)	n (%)	n (%)			n (%)	n (%)			n (%)	n (%)		
	4051 (100.00)	283 (6.99)	3768 (93.01)			738 (18.22)	3313 (81.78)			1736 (42.85)	2315 (47.15)		
**BMI**					<0.001 ***				<0.001 ***				<0.001 ***
Normal weight ^Ref.^	2097 (51.76)	144 (6.87)	1953 (93.13)			414 (19.74)	1683 (80.26)			956 (45.59)	1141 (54.41)		
Underweight	487 (12.02)	41 (8.42)	446 (91.58)	1.60 (1.21–2.12)	0.001 **	151 (31.01)	336 (68.99)	1.79 (1.44–2.24)	<0.001 ***	269 (55.24)	218 (44.76)	1.44 (1.18–1.76)	<0.001 ***
Overweight/Obesity	1467 (36.21)	98 (6.68)	1369 (93.32)	0.74 (0.51–1.08)	0.116	173 (11.79)	1294 (88.21)	0.64 (0.52–0.78)	<0.001 ***	511 (34.83)	956 (65.17)	0.71 (0.62–0.82)	<0.001 ***
**GWG**					0.465				<0.001 ***				<0.001 ***
Optimal ^Ref.^	1347 (33.25)	81 (6.02)	1266 (93.98)			209 (15.52)	1138 (84.48)			561 (41.65)	786 (58.35)		
Below	1715 (42.34)	157 (9.15)	1558 (90.85)	1.22 (0.85–1.75)	0.293	422 (24.61)	1293 (75.39)	1.66 (1.38–2.00)	<0.001 ***	846 (49.33)	869 (50.67)	1.29 (1.11–1.49)	0.001 **
Above	989 (24.41)	45 (4.55)	944 (95.45)	1.14 (0.87–1.50)	0.356	107 (10.82)	882 (89.18)	0.74 (0.58–0.95)	0.019 *	329 (33.27)	660 (66.73)	0.75 (0.63–0.90)	0.001 **

* *p* < 0.05, ** *p* < 0.01, *** *p* < 0.001, ^Ref.^: reference category.

## Data Availability

The datasets generated and/or analyzed during the current study are not publicly available due to privacy and ethical restrictions. Requests for access to the data may be directed to Rachadaporn Jantasuwan.
